# The efficacy of artesunate in animal models of sepsis: a systematic review and meta-analysis

**DOI:** 10.3389/fphar.2026.1748083

**Published:** 2026-01-28

**Authors:** Wenzhan Xie, Linxi Lv, Tian Wang, Jialong Wei, Yanshan Gui, Bing Han, Laixian Zhou, Hui Feng, Wei Gu

**Affiliations:** 1 Center of Smart Laboratory and Molecular Medicine, School of Medicine, Chongqing University, Chongqing, China; 2 Department of Nephrology, First Affiliated Hospital, Army Medical University, Chongqing, China

**Keywords:** artesunate, sepsis, animal models, systematic review, meta-analysis

## Abstract

**Background:**

Sepsis is a life-threatening condition caused by a dysregulated host response to infection, characterized by biphasic immune dysregulation and high mortality rates. Artesunate (AS), a semisynthetic artemisinin derivative, has demonstrated broad pharmacological properties, yet its overall efficacy and mechanisms in sepsis remain systematically unassessed at the preclinical level.

**Objectives:**

In this study, we aimed to conduct the first systematic review and meta-analysis to evaluate the therapeutic efficacy and underlying mechanisms of AS in animal models of sepsis.

**Methods:**

We systematically searched five electronic databases up to 3 September 2025, for controlled *in vivo* studies analyzing the effects of AS in septic animals. The study quality was assessed using the SYRCLE risk-of-bias tool, and evidence certainty was rated via the GRADE approach. Statistical analyses, including meta-analysis, publication bias, and sensitivity analyses, were performed using RevMan 5.4 and Stata 17.0.

**Results:**

Fifteen studies involving mice and rats were included. Meta-analysis indicated that AS was associated with improved survival (10 studies, OR: 6.87, 95% CI: 3.81–12.41, *p* < 0.00001), reduced bacterial load, and promotion of body weight recovery. Organ protection was evidenced by attenuated lung injury (reduced histological scores, MPO activity, and wet-to-dry ratio) and improved liver function (decreased AST and ALT levels). Analysis of cytokine data from different time-points suggested a potential phase-dependent immunomodulatory effect: AS suppressed pro-inflammatory cytokines (TNF-α and IL-6) during the hyperinflammatory phase while restoring immune competence in the immunosuppressive phase, accompanied by elevated IL-1β. Furthermore, AS reduced apoptosis (decreased TUNEL-positive cells) and enhanced pro-survival signaling (increased p-mTOR/mTOR ratio); however, its effect on caspase-3 was not significant. Sensitivity analyses supported the robustness of the primary findings, and no significant publication bias was detected within the limits of the available studies.

**Conclusion:**

AS is associated with survival benefits and multi-organ protection in septic animal models through multimodal mechanisms, potential phase-aware immunomodulation, antiapoptotic effects, and enhanced bacterial clearance. Despite methodological heterogeneity across studies, these preclinical findings support further investigation of AS as a potential therapeutic candidate for sepsis treatment.

**Systematic Review Registration:**

https://www.crd.york.ac.uk/PROSPERO/view/CRD420251146068.

## 1 Introduction

Sepsis is a life-threatening organ dysfunction triggered by a dysregulated host response to infection (Singer et al., 2016). It represents a major global healthcare challenge, with an annual incidence exceeding 49 million and causing approximately 11 million deaths worldwide (Rudd et al., 2020). Despite advances in antimicrobial and supportive therapies, the mortality rate remains high at 30%–40%, and its incidence has continued to rise over the past three decades (Stoller et al., 2016; Rhee et al., 2017; Álvaro-Meca et al., 2018). The core pathological process of sepsis is highly dynamic, with its immune status being particularly complex. Extensive evidence confirms that the early phase is typified by cytokine storm-induced immune hyperactivation, whereas massive immune-cell death and exhaustion drive a shift to immune suppression in the late phase (Hotchkiss et al., 2013; van der Poll et al., 2017). Clinically, early immune hyperactivation is the main cause of initial sepsis mortality, and immune suppression drives late-stage deaths. Although anti-inflammatory therapies help most patients pass the hyperactivation phase, mortality remains 40%–80% in those progressing to suppression (Hotchkiss and Karl, 2003; Angus and van der Poll, 2013). To date, no targeted therapy has successfully addressed the biphasic and multifactorial nature of sepsis, underscoring the urgent need for multimodal treatment strategies aligned with its dynamic progression (van der Poll et al., 2017; Pierrakos et al., 2020).

The search for novel sepsis treatments has involved the evaluation of various natural compounds, such as resveratrol (Zhou et al., 2019) and curcumin (Zhang et al., 2025), which primarily confer anti-inflammatory and antioxidant activities. However, their therapeutic potential is often constrained by a limited capacity to adapt to the dynamic immune landscape of sepsis. In this context, artemisinin and its derivatives have gained considerable attention due to their broader range of pleiotropic properties. Artesunate (AS, C19H28O8, Supplementary Figure S1), a water-soluble semisynthetic derivative of artemisinin, a sesquiterpene lactone isolated from *Artemisia annua L*., has emerged as a compelling candidate for sepsis treatment (Kong and Tan, 2015; Yao et al., 2022). AS offers several translational advantages over artemisinin, including improved chemical stability, enhanced oral bioavailability, and a well-established clinical safety profile (Shi et al., 2022). Beyond its recognized efficacy against severe and cerebral malaria, AS exhibits a broad spectrum of pleiotropic pharmacological properties, such as anti-inflammatory, neuroprotective, antitumor, and antiapoptotic activities (Jiang et al., 2011; Meng et al., 2018; Wang et al., 2020; Ruwizhi et al., 2022). Notably, recent preclinical evidence has highlighted its therapeutic potential in sepsis, demonstrating an ability to modulate multiple key pathophysiological pathways involved in the disorder, such as the pro-inflammatory nuclear factor-kappa B (NF-κB) and NOD-like receptor pyrin domain containing 3 (NLRP3) inflammasome signaling (Liu et al., 2023), cell survival-related phosphatidylinositol 3-kinase (PI3K)/Ak strain transforming (Akt)/mechanistic target of rapamycin (mTOR) axis (Zhang et al., 2020), and the antioxidant nuclear factor erythroid 2-related factor 2 (Nrf2)/heme oxygenase-1 (HO-1) pathway (Zhang et al., 2020). A key advantage of AS is its phase-dependent ¥ immunomodulatory capability, which mirrors the biphasic profile of sepsis while effectively controlling excessive inflammation and preventing immune exhaustion. Moreover, AS has been shown to inhibit lymphocyte apoptosis, promote autophagic flux, and mitigate multi-organ damage, further supporting its potential as a multifaceted therapeutic agent against the systemic manifestations of sepsis.

Given the growing body of preclinical studies assessing the therapeutic role of AS in sepsis, a systematic synthesis and comparative assessment of the available evidence is urgently needed to prioritize its clinical translation among other candidate natural compounds. Although animal models offer valuable insights into disease mechanisms and treatment potential (Sena et al., 2014), translating these findings into clinical practice remains a considerable challenge. Meta-analyses of animal studies can clarify the magnitude and consistency of treatment effects, identify potential sources of heterogeneity, and provide foundational evidence for designing future clinical trials (Vesterinen et al., 2014; Spanagel, 2022). To date, no systematic review or meta-analysis has comprehensively evaluated the efficacy and underlying mechanisms of AS in animal models of sepsis. In this study, we therefore aim to address this gap by conducting the first systematic review and meta-analysis to assess the therapeutic potential of AS in septic animal models, with the objective of establishing a robust preclinical evidence base to inform subsequent research and potential clinical translation.

## 2 Methods

This study adhered to the Preferred Reporting Items for Systematic Review and Meta-Analysis (PRISMA) guidelines and was registered with PROSPERO (CRD420251146068).

### 2.1 Search strategy

A systematic search of five electronic databases (PubMed, Web of Science, The Cochrane Library, Scopus, and Embase) was conducted up to 3 September 2025 using a combination of MeSH terms and free-text keywords related to “sepsis” and “artesunate” (Supplementary Table S1). The reference lists of relevant articles were manually screened.

### 2.2 Eligibility criteria

Studies were included based on the PICOS framework, where, P (population): *in vivo* septic animal models; I (intervention): AS intervention; C (control): vehicle, saline, or no treatment; O (outcomes) primary outcome: survival rate, secondary outcomes: colony-forming unit (CFU), body weight, serum levels of inflammatory cytokines [tumor necrosis factor-alpha (TNF-α), interleukin-6 (IL-6), and interleukin-1 beta (IL-1β)], markers of organ injury [aspartate aminotransferase (AST), alanine aminotransferase (ALT), lung injury score, myeloperoxidase (MPO) activity, and wet-to-dry (W/D) ratio], and apoptosis-related biomarkers [terminal deoxynucleotidyl transferase dUTP nick end labeling (TUNEL)-positive cells, the ratio of phosphorylated mechanistic target of rapamycin to total mTOR (p-mTOR/mTOR), and caspase-3 expression]; S (study design): controlled animal studies.

The exclusion criteria were as follows: 1. the use of combined therapies that could confound the results; 2. incomplete or unavailable data for meta-analysis; 3. secondary literature types (reviews, abstracts, *etc*.); and 4. duplicate publications, with only the most complete dataset retained.

### 2.3 Data extraction and quality assessment

Two researchers independently extracted data (publication details, animal characteristics, modeling method, AS administration, and outcomes) and resolved discrepancies with a third reviewer. Original data from figures were extracted by contacting authors or via WebPlotDigitizer. In studies involving multiple intervention doses or time-points—resulting in several datasets for the same outcome—the dataset corresponding to the most effective dose or time-point was selected for meta-analysis. Methodological quality was assessed using the Systematic Review Centre for Laboratory Animal Experimentation (SYRCLE) RoB tool (for animal studies) (Hooijmans et al., 2014) and Grading of Recommendations Assessment, Development, and Evaluation (GRADE) approach (https://gdt.gradepro.org/app/) (Guyatt et al., 2008; Balshem et al., 2011). Two reviewers conducted assessments independently, with discrepancies resolved via discussion.

### 2.4 Statistical analysis

The meta-analysis was conducted using Review Manager 5.4 (Cochrane Collaboration, Oxford, UK) and Stata 17.0 (StataCorp LLC) software. Based on data types, the corresponding effect measures were applied. For dichotomous outcomes, odds ratios (ORs) with 95% confidence intervals (CIs) were calculated and pooled using the Mantel–Haenszel method. For continuous variables, mean differences (MD) with 95% CIs were computed. A *p*-value < 0.05 was considered statistically significant. Heterogeneity among studies was assessed using the chi-square test (χ^2^) and I^2^ statistic. A fixed-effect model was applied when *p* > 0.1 and I^2^ < 50% (Pereira et al., 2010; Thorlund et al., 2012; Hoaglin, 2016), indicating low heterogeneity; otherwise, a random-effects model was used (Borenstein et al., 2010). Publication bias of the included studies was evaluated using a funnel plot, Egger’s test (Egger et al., 1997), and the trim-and-fill method (Peters et al., 2007) based on standard error (Lau et al., 2006) when >9 studies were included for a single outcome measure. We performed sensitivity analyses in outcomes with more than two studies to investigate the influence of individual studies on the overall pooled effect size.

## 3 Results

**FIGURE 1:**
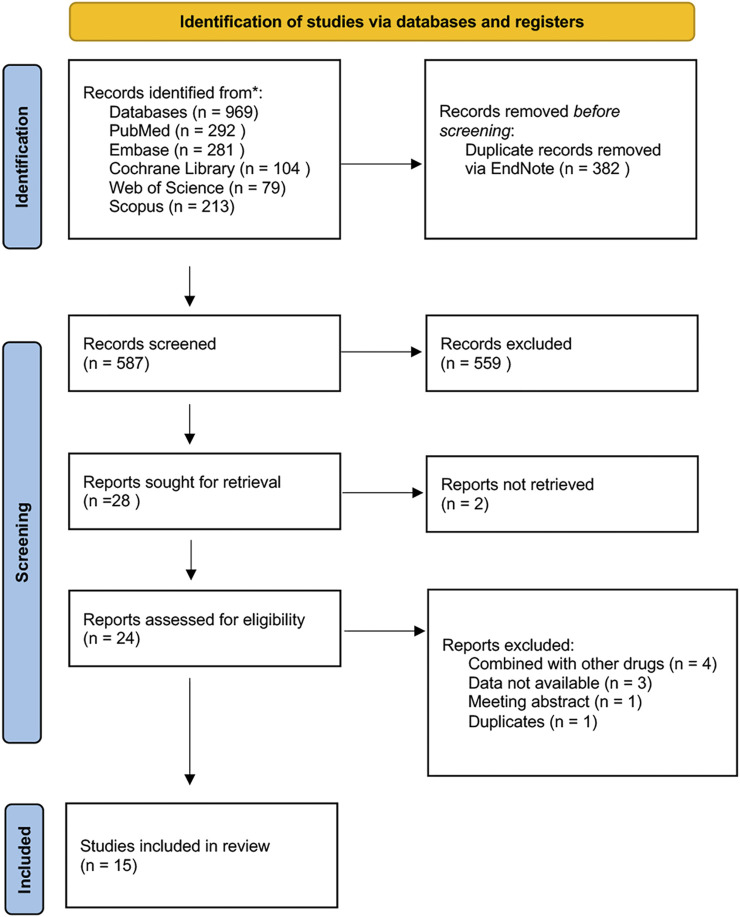
Flow diagram for the study selection process.

Based on the search strategy, a total of 969 studies were identified. After removing duplicates, 587 studies remained. Following further exclusion, 15 articles were included in the meta-analysis. The study selection process, along with reasons for exclusion, is depicted in Figure 1.

### 3.1 Quality appraisal

**FIGURE 2:**
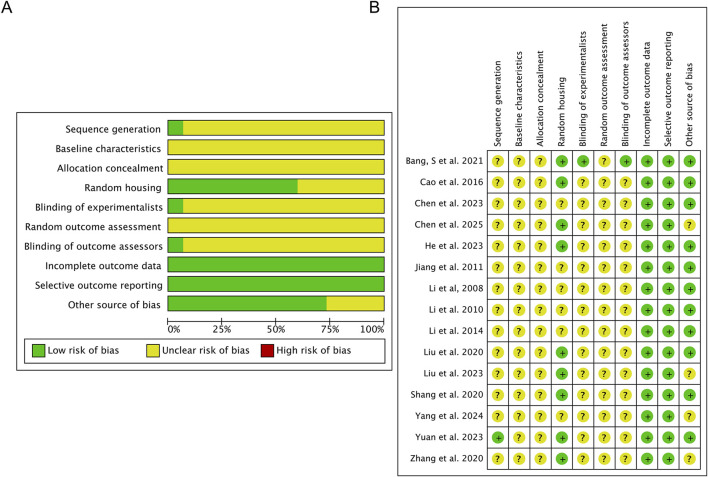
Risk-of-bias assessment of the 15 studies included in this meta-analysis based on SYRCLE’s risk-of-bias tool: **(A)** risk-of-bias graph and **(B)** risk-of-bias summary.

The methodological quality of the 15 included studies was assessed using the SYRCLE risk-of-bias tool, with the detailed results visualized in Figure 2 and summarized in Supplementary Table S2. Reporting of key methodological details was generally incomplete. Only one study adequately described random sequence generation, and none reported sufficient information regarding allocation concealment. Blinding of personnel and outcome assessors was explicitly stated in only one study, and no study described random housing or blinding during outcome assessment. While all studies reported basic animal characteristics, none provided baseline data for specific outcome measures. All studies were considered to be at low risk for incomplete outcome data and selective reporting. Nevertheless, the widespread unclear or high risk of bias in critical domains such as allocation concealment and blinding underscores substantial methodological limitations in the available evidence. These shortcomings led to a reduction in the overall certainty of evidence in the GRADE assessment, which was predominantly rated as low or moderate for critical outcomes (Supplementary Table S3).

### 3.2 Study characteristics

The basic characteristics of the included studies are summarized in Table 1. The relevant publications were issued between 2008 and 2025, with sample sizes ranging from 20 to 168. The sepsis animal models in the included studies were primarily induced by cecal ligation and puncture (CLP) or intraperitoneal injection of lipopolysaccharide (LPS), whereas some studies utilized bacterial injection or a combination of methods. The animals used included various strains of mice and rats. Regarding the intervention, the effective dose of AS ranged from 5 to 300 mg/kg. Administration was predominantly via the intraperitoneal route (7/15 studies), with the remaining studies using intravenous, intramuscular, intratracheal, or oral routes. The timing of intervention varied relative to sepsis induction, including pretreatment, concurrent administration, and *post hoc* treatment at time-points ranging from immediately to after 72 h. These variabilities are critical considerations when interpreting the pooled results and their translational implications. All studies reported sepsis-related outcomes, with survival rate and systemic inflammatory cytokine levels being the most frequently assessed parameters.

**TABLE 1:** Summary characteristics of the studies included in the review.

Study	Species	Sepsis model	AS dose (mg/kg)	AS route	Time of AS administration relative to model induction	Control	Organ	Outcome	Refs.
Chen et al. (2025) China	C57BL/6 mice (6–8 weeks, male)	CLP	300	p.o.	Pre-CLP (5 days) + Post-CLP (3 days)	1% CMC	Kidney	• Body weight • Kidney index • Serum SCr • Serum BUN • p-AMPK/LC3-II/p-mTOR protein and mRNA levels	Chen et al. (2025)
Yang et al. (2024) China	C57BL/6 mice (6–8 weeks, 18 g–22 g)	LPS (5 mg/kg)	5	i.p.	Concurrent with LPS	LPS	Liver	• Serum AST • Serum ALT • MALAT1 mRNA levels • IFIH1/STAT1 protein levels	Yang et al. (2024)
Yuan et al. (2023) China	BALB/c mice (6–8 weeks, male, 20 g–24 g)	CLP and PA (1.5 × 10^8^ CFU/kg)	10	i.m.	Post-CLP (22, 26, 48, and 72 h)	NS	Whole body	• Survival rate • Blood/lungs/spleen PA Loads • Serum/lungs/spleen TNF-α • Serum/lungs/spleen IL-6 • Serum/lungs/spleen IL-1β • Caspase-3/cleaved Caspase-3/Caspase-9/cleaved Caspase-9/ERK/p-ERK protein levels	Yuan et al. (2023)
Liu et al. (2023) China	SD rats (250 g–300 g)	LPS (5 mg/kg)	25	i.p.	Post-LPS (1 h)	PBS	Lung	• Lung injury scores • MPO activity • W/D weight ratio • SIRT1 mRNA levels • TUNEL-positive cells	Liu et al. (2023)
He et al. (2023) China	BALB/c mice (6–8 weeks, male, 18 g–22 g)	CLP	10	i.p.	Post-CLP (4 h)	NS	Liver	• Survival rate • Serum AST • Serum ALT • Serum TNF-α • Serum IL-6 • Serum IL-1β • Serum IFN-γ • PD-1/PD-L1 protein levels	He et al. (2023)
Chen et al. (2023) China	BALB/c mice (6–8 weeks, male, 20 g–22 g)	CLP	10	i.p.	Post-CLP (4 h)	NS	Spleen	• Survival rate • Body weight • Spleen index/CAT/SOD/GSH/NO/MDA	Chen et al. (2023)
Bang, S et al. (2021) USA	C57BL/6 mice (8–10 weeks, male and female)	LPS (10 mg/kg)	10	i.p.	Concurrent with LPS	PBS	Whole body	• Survival rate • Body temperature	Bang et al. (2021)
Zhang et al. (2020) China	SD rats (220 g–250 g)	LPS (5 mg/kg)	15	i.t.	Post-LPS (1 h)	PBS	Lung	• Lung injury scores • Wet/dry weight ratio • BALF TNF-α • BALF IL-6 • MPO activity • Serum MDA • Serum SOD • Serum GSH-Px • Cleaved Caspase-3/Caspase-3/p-mTOR/MTOR/AKT/p-AKT/PI3K/p-PI3K protein levels • TUNEL apoptotic index • TUNEL positive cells field	Zhang et al. (2020)
Liu et al. (2020) China	BALB/c (6–8 weeks, male, 18 g–22 g)	LPS (50 mg/kg) or *S. aureus* (5.0 × 10^6^ CFU/kg)	10	i.p.	Post-LPS	NS	Spleen	• Serum/peritoneal fluid TNF-α • Blood/spleen/lung CFU • LC3-II/LC3-I protein levels	Liu et al. (2020)
Shang et al. (2020) China	BALB/c (6–8 weeks, half male and half female, 18 g–22 g)	CLP and PA (2.5 × 10^9^ CFU/kg)	10	i.m.	Post-CLP (22, 26, 48, and 72 h)	Non-operation	Whole body	• Mortalities rate • Serum/Spleen/Lungs TNF-α • Serum/Spleen/Lungs IL-6 • Serum/Spleen/Lungs IL-1β • Blood/Spleen/Lungs PA Loads • ATG16L1、ATG5 protein levels • VDR protein and mRNA levels	Shang et al. (2020)
Cao et al. (2016) China	KM mice (6–8 weeks, male, 20 g–22 g)	CLP	15	i.p.	Pre-CLP (1 h)	Saline	Lung	• Survival rate • Lung injury scores • W/D weight ratio • Serum TNF-α • Serum IL-6 • BALF TNF-α • BALF IL-6 • MPO activity • COX-2/iNOS/HO-1 protein and mRNA levels • NF-κB/Nrf2 protein levels	Cao et al. (2016)
Li et al. (2014) China	KM mice (4–6 weeks, male and female, 18 g–20 g)	CLP	30	i.m.	Post-CLP (immediately)	Non-operation	Liver	• Survival rate • Serum TNF-α • Serum IL-6 • Serum LPS • Serum AST • Serum ALT	Li et al. (2014)
Jiang et al. (2011) China	KM mice (male and female, 18 g–22 g)	WHO-2 (6.0 × 10^9^ CFU/mL)	30	i.v.	Post-WHO-2 (4 h, then twice daily for 7 days)	NS	Whole body	• Survival rate • Serum TNF-α • Serum IL-6 • CFU	Jiang et al. (2011)
Li et al. (2010) China	KM mice (male and female, 18 g–22 g)	Heat-killed *S. aureus* (1.6 × 10^12^ CFU/kg)	30	i.v.	Concurrent + Post (4, 24, and 48 h)	NS	Whole body	• Survival rate • Serum TNF-α	Li et al. (2010)
Li et al. (2008) China	KM mice (4–8 weeks, male and female, 19.4 g ± 1.8 g)	Heat-killed *E. coli* (1.25 × 10^11^ CFU/kg)	45	i.v.	Concurrent + Post (0, 4, 24, and 48 h)	NS	Whole body	• Survival rate • Serum endotoxin • Serum TNF-α	Li et al. (2008)

Abbreviations: AKT, protein kinase B; ALT, alanine aminotransferase; AMPK, AMP-activated protein kinase; AST, aspartate aminotransferase; ATG16L1, autophagy-related protein 16–1; ATG5, autophagy-related protein 5; BALF, bronchoalveolar lavage fluid; BUN, blood urea nitrogen; CAT, catalase; Caspase-3, cysteinyl aspartate-specific proteinase-3; CFU, colony-forming unit; CLP, cecal ligation and puncture; COX-2, cyclooxygenase-2; ERK, extracellular signal-regulated kinase; GSH, glutathione; GSH-Px, glutathione peroxidase; HO-1, heme oxygenase-1; IFN-γ, interferon gamma; IFIH1, interferon induced with helicase C domain 1; IL-1β, interleukin-1 beta; IL-6, interleukin-6; i.m., intramuscular injection; iNOS, inducible nitric oxide synthase; i.p., intraperitoneal injection; i.t., intratracheal instillation; i.v., tail vein injection; LC3-II, microtubule-associated proteins 1A/1B light chain 3B-II; LPS, lipopolysaccharide; MALAT1, metastasis-associated lung adenocarcinoma transcript 1; MDA, malondialdehyde; MPO, myeloperoxidase; mTOR, mammalian target of rapamycin; NF-κB, nuclear factor kappa B; NO, nitric oxide; Nrf2, nuclear factor erythroid 2-related factor 2; p-AKT, phospho-protein kinase B; p-AMPK, phospho-AMP-activated protein kinase; PA, *Pseudomonas aeruginosa*; PD-1, programmed cell-death protein 1; PD-L1, programmed death-ligand 1; P-ERK, phospho-ERK; PI3K, phosphoinositide 3-kinase; p-PI3K, phospho-phosphoinositide 3-kinase; p.o., oral gavage; p-mTOR, phospho-mammalian target of rapamycin; SCr, serum creatinine; SIRT1, sirtuin 1; SOD, superoxide dismutase; STAT1, signal transducer and activator of transcription 1; TUNEL, terminal deoxynucleotidyl transferase dUTP nick end labeling; VDR, vitamin D receptor; W/D, wet/dry weight ratio.

## 4 Quantitative synthesis (meta-analysis)

### 4.1 General efficacy outcomes

**FIGURE 3:**
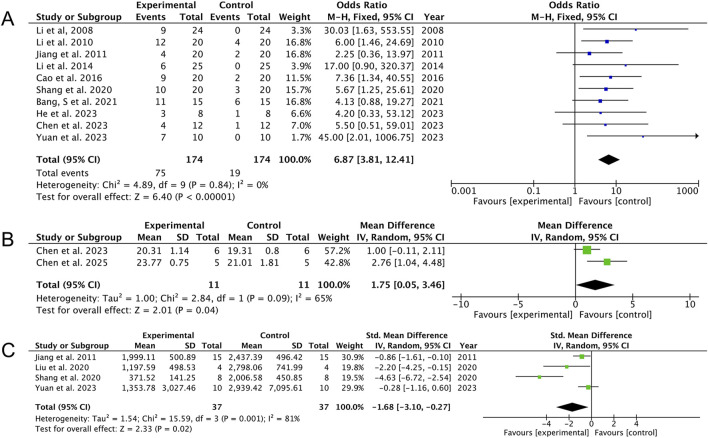
Forest plots of the effects of artesunate vs. control on general efficacy. **(A)** Survival rate, **(B)** body weight, and **(C)** CFU.

The analysis of ten studies (Li et al., 2008; Li et al., 2010; Jiang et al., 2011; Li et al., 2014; Cao et al., 2016; Shang et al., 2020; Bang et al., 2021; Chen et al., 2023; He et al., 2023; Yuan et al., 2023) provides evidence that AS improved survival in septic animals compared with the controls [OR: 6.87 (95% CI: 3.81, 12.41), *p* < 0.00001; heterogeneity: I^2^ = 0%, *p* = 0.84, GRADE of evidence: moderate; Figure 3A]. Data from two studies (Chen et al., 2023; Chen et al., 2025) indicated an increase in body weight associated with AS treatment [MD: 1.75 (95% CI: 0.05, 3.46), *p* = 0.04; heterogeneity: I^2^ = 65%, *p* = 0.09, GRADE of evidence: low; Figure 3B]. Furthermore, a decrease in bacterial load was observed across four studies (Jiang et al., 2011; Liu et al., 2020; Shang et al., 2020; Yuan et al., 2023) [MD: −1.68 (95% CI: −3.10, −0.27), *p* = 0.02; heterogeneity: I^2^ = 81%, *p* = 0.001, GRADE of evidence: low; Figure 3C].

### 4.2 Lung injury

**FIGURE 4:**
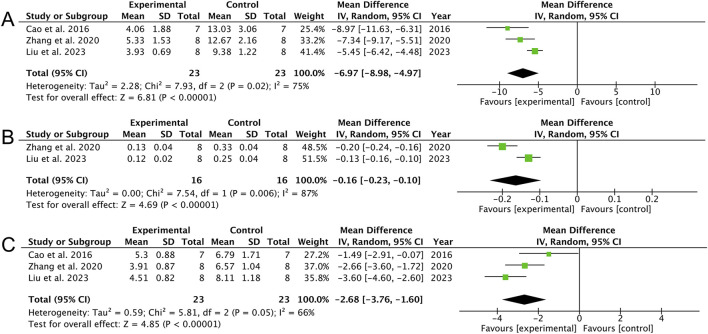
Forest plots of the effects of artesunate vs. control on lung injury. **(A)** Lung injury scores, **(B)** MPO activity, and **(C)** W/D ratio.

Meta-analysis of three studies (Cao et al., 2016; Zhang et al., 2020; Liu et al., 2023) indicates that AS attenuated lung injury, as evidenced by reduced histological scores [MD: −6.97 (95% CI: −8.98, −4.97), *p* < 0.00001; heterogeneity: I^2^ = 75%, *p* = 0.02, GRADE of evidence: low; Figure 4A]. Data from two studies (Zhang et al., 2020; Liu et al., 2023) showed a decrease in pulmonary myeloperoxidase activity [MD: −0.16 (95% CI: −0.23, −0.10), *p* < 0.00001; heterogeneity: I^2^ = 87%, *p* = 0.006, GRADE of evidence: low; Figure 4B]. Furthermore, analysis of three studies (Cao et al., 2016; Zhang et al., 2020; Liu et al., 2023) suggests a reduction in the wet-to-dry ratio [MD: −2.68 (95% CI: −3.76, −1.60), *p* < 0.00001; heterogeneity: I^2^ = 66%, *p* = 0.05, GRADE of evidence: moderate; Figure 4C].

### 4.3 Liver injury

**FIGURE 5:**
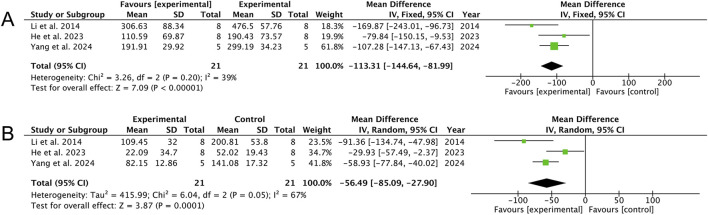
Forest plots of the effects of artesunate vs. control on liver function. **(A)** AST and **(B)** ALT levels.

AS treatment appears to confer hepatoprotection, as shown by a marked reduction in serum aspartate aminotransferase levels across three studies (Li et al., 2014; He et al., 2023; Yang et al., 2024) [MD: −113.31 (95% CI: −144.64, −81.99), *p* < 0.00001; heterogeneity: I^2^ = 39%, *p* = 0.20, GRADE of evidence: moderate; Figure 5A]. Similarly, a decrease in alanine aminotransferase levels was observed in the same three studies [MD: −56.49 (95% CI: −85.00, −27.90), *p* = 0.0001; heterogeneity: I^2^ = 67%, *p* = 0.05, GRADE of evidence: moderate; Figure 5B].

### 4.4 Inflammatory cytokines

**FIGURE 6:**
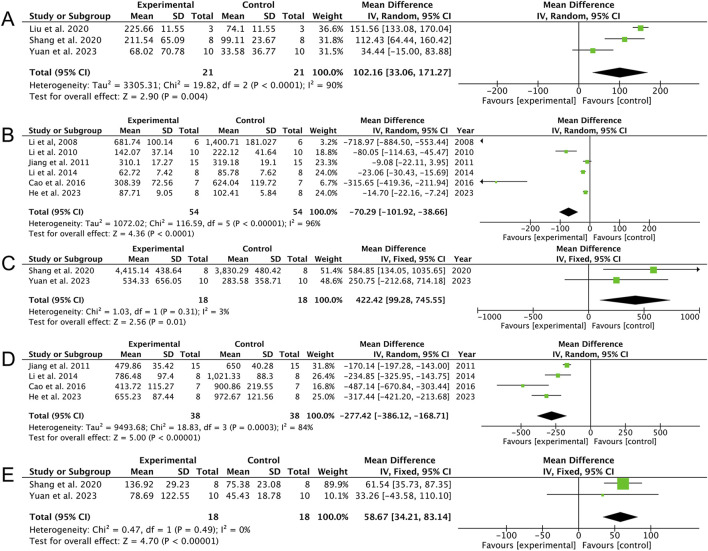
Forest plots of the effects of artesunate vs. control on serum inflammatory cytokines. **(A)** TNF-α (up), **(B)** TNF-α (down), **(C)** IL-6 (up), **(D)** IL-6 (down), and **(E)** IL-1β (up).

When synthesizing data grouped by sampling time-points, a pattern of phase-dependent regulation was retrospectively inferred. AS demonstrated bidirectional modulation of systemic inflammation. During the late immunosuppressive phase, AS restored circulating levels of TNF-α [three studies (Liu et al., 2020; Shang et al., 2020; Yuan et al., 2023); MD: 102.16 (95% CI: 33.06, 171.27), *p* = 0.004; heterogeneity: I^2^ = 90%, *p* < 0.00001, GRADE of evidence: low; Figure 6A] and IL-6 [two studies (Shang et al., 2020; Yuan et al., 2023); MD: 422.42 (95% CI: 99.28, 745.55), *p* = 0.01; heterogeneity: I^2^ = 3%, *p* = 0.31, GRADE of evidence: moderate; Figure 6C]. Conversely, during the early hyperinflammatory phase, it suppressed the levels of both TNF-α [six studies (Li et al., 2008; Li et al., 2010; Jiang et al., 2011; Li et al., 2014; Cao et al., 2016; He et al., 2023); MD: −70.29 (95% CI: −101.92, −38.68), *p* < 0.00001; heterogeneity: I^2^ = 96%, *p* < 0.00001, GRADE of evidence: low; Figure 6B] and IL-6 [four studies (Jiang et al., 2011; Li et al., 2014; Cao et al., 2016; He et al., 2023); MD: −227.42 (95% CI: −386.12, −168.71), *p* < 0.00001; heterogeneity: I^2^ = 84%, *p* = 0.0003, GRADE of evidence: low; Figure 6D]. Additionally, AS treatment elevated IL-1β levels across two studies (Shang et al., 2020; Yuan et al., 2023) [MD: 58.67 (95% CI: 34.21, 83.14), *p* < 0.00001; heterogeneity: I^2^ = 0%, *p* = 0.49, GRADE of evidence: moderate; Figure 6E]. Additionally, although one study (He et al., 2023) reported a reduction in IL-1β levels with AS treatment, meta-analysis was not performed due to the inclusion of only a single study. Analyses of cytokine levels and bacterial load in the lung and spleen tissues (Supplementary Figures) further support the time-dependent immunomodulatory effects and effective clearance of local infection by AS.

### 4.5 Apoptosis-related biomarkers

**FIGURE 7:**
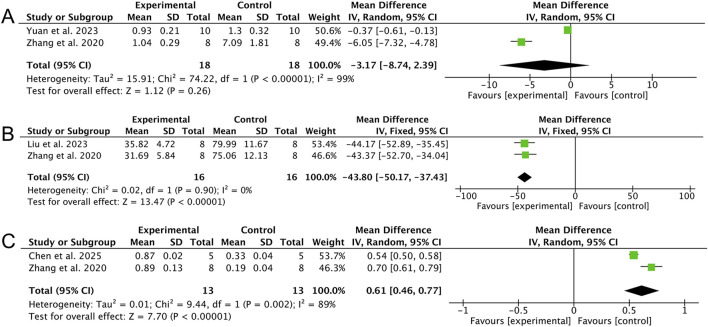
Forest plots of the effects of artesunate on apoptosis-related biomarkers. **(A)** Caspase-3 expression, **(B)** TUNEL-positive cells, and **(C)** p-mTOR/mTOR ratio.

Meta-analysis of apoptosis-related biomarkers provides preliminary evidence that AS attenuated apoptosis in septic models. AS treatment markedly reduced the number of apoptotic cells [two studies (Zhang et al., 2020; Liu et al., 2023), MD: −43.80 (95% CI: −50.17, −37.43), *p* < 0.00001; heterogeneity: I^2^ = 0%, p = 0.90, GRADE of evidence: moderate; Figure 7B] and enhanced the phosphorylation of the pro-survival regulator mTOR, as indicated by an increased p-mTOR/mTOR ratio [two studies (Zhang et al., 2020; Chen et al., 2025), MD: 0.61 (95% CI: 0.46, 0.77), *p* < 0.00001; heterogeneity: I^2^ = 89%, GRADE of evidence: very low; Figure 7C]. In contrast, the overall effect on caspase-3 expression was not statistically significant [two studies (Zhang et al., 2020; Yuan et al., 2023), MD: −3.17 (95% CI: −8.74, 2.39), *p* = 0.26, GRADE of evidence: very low; Figure 7A], with substantial heterogeneity (I^2^ = 99%) that is likely attributable to methodological variations in protein normalization.

### 4.6 Publication bias and sensitivity analysis

**FIGURE 8:**
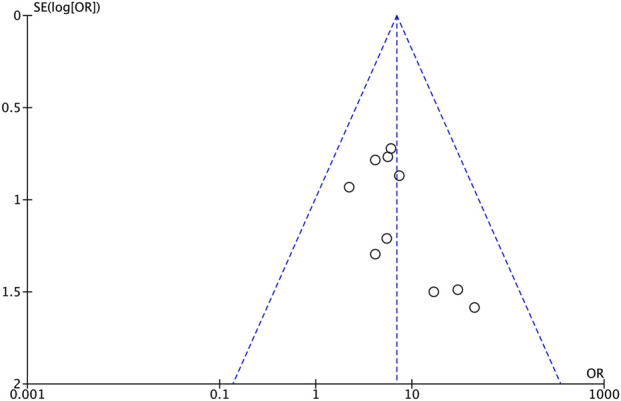
Funnel plot of survival rates.

All included studies involved the use of AS for treating septic animal models. Using survival outcomes as the main variable, the included studies were evaluated for the effect of the study size. The funnel plot was largely symmetrical (Figure 8). Although Egger’s test was not statistically significant (p = 0.053, Table 2), the statistical power of both the funnel plot and Egger’s test is insufficient due to the small number of included studies, and thus, publication bias cannot be fully excluded. Given its marginal significance and to preclude any potential bias, a trim-and-fill analysis was conducted. This analysis imputed two missing studies, and the adjusted effect size (HR = 1.646) was nearly identical to the original (HR = 1.807) and remained highly significant (Table 3), indicating that the primary survival outcome is relatively robust despite the inability to rule out publication bias.

**TABLE 2:** Egger’s publication bias of survival rates.

Item	No. studies	Pooled effect size	95% confidence interval	P-value	Heterogeneity (I^2^, P-value)
Before trim and fill	10	1.807	1.196 to 2.419	<0.001	0.0%, P = 0.856
After trim and fill	12	1.646	1.059 to 2.234	<0.001	0.0%, P = 0.690

**TABLE 3:** Egger’s test and trim-and-fill analysis for survival rates.

Standard effect	Coefficient	Standard error	t	P-value	95% Confidence interval
Slope	0.3104039	0.684204	0.45	0.662	−1.267373 to 1.888181
Bias	1.57736	0.6934295	2.27	0.053	−0.0216909 to 3.176412

**FIGURE 9:**
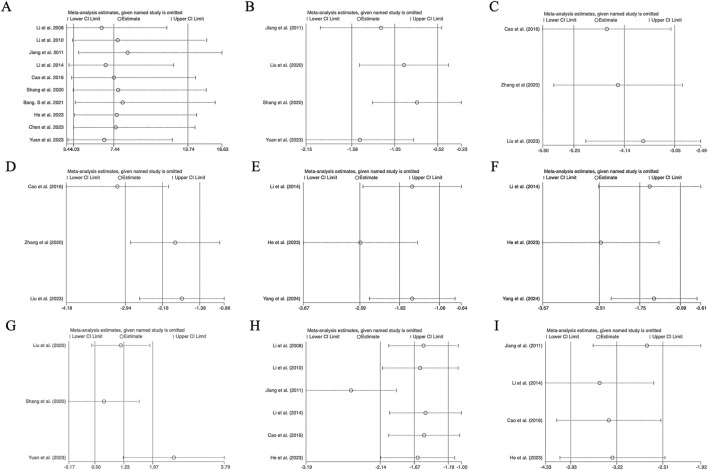
Sensitivity analysis chart. **(A)** Survival rate, **(B)** CFU, **(C)** lung injury scores, **(D)** W/D ratio, **(E)** AST levels, **(F)** ALT levels, **(G)** TNF-α (up), **(H)** TNF-α (down), and **(I)** IL-6 (down).

The results of the sensitivity analysis were evaluated by systematically excluding individual studies and comparing the recalculated pooled effect sizes with the overall estimates. As shown in Figure 9, no significant differences were observed between the recalculated results and the original overall results across all outcome measures. This consistency indicates that the findings of our study are robust and are not unduly influenced by any single dataset.

## 5 Discussion

### 5.1 Summary of evidence

This meta-analysis provides preliminary evidence that AS improves survival and exerts multidimensional protection in septic animal models. AS was associated with treatment-enhanced survival rates while reducing bacterial load and promoting body weight recovery. The agent attenuated multiple organ injuries, evidenced by improved lung histopathology and liver function biomarkers. Furthermore, analysis stratified by time-point suggested that AS exerts phase-dependent immunomodulation, being associated with the suppression of early hyperinflammation and potential restoration of immune function in later stages, accompanied by reduced apoptosis and enhanced autophagy. For the primary survival outcome, publication bias assessment did not show statistically significant evidence of bias, but the small number of included studies led to insufficient statistical power for the funnel plot and Egger’s test; thus, publication bias cannot be fully excluded. Sensitivity analysis demonstrated that no individual study unduly influenced the overall effect estimates.

### 5.2 Mechanism overview

**FIGURE 10:**
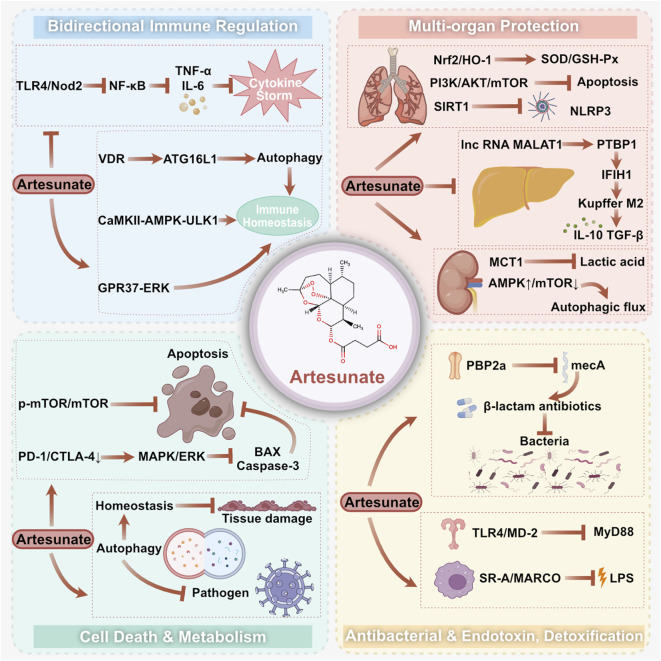
Schematic representation of the potential molecular mechanism of artesunate inhibition of sepsis.

AS exerts its therapeutic efficacy against sepsis through a multimodal mechanism. The following synthesis distinguishes between effects supported by the present meta-analysis and deeper mechanistic insights that are primarily derived from individual preclinical reports. Although the latter provide valuable hypotheses for specific molecular pathways, they require further independent replication and validation. This integrated perspective encompasses phase-dependent immunomodulation, organ-protective effects, regulation of apoptosis and autophagy, and direct antibacterial and endotoxin-clearing actions (Figure 10).

#### 5.2.1 Immunomodulation and homeostatic reconstitution

Our meta-analysis, by grouping outcomes based on sampling time-points, suggests that AS may possess phase-dependent immunomodulatory capabilities. This intriguing possibility, which involves suppressing early hyperinflammation while potentially restoring function later, requires consideration as a hypothesis generated from the extant data pattern. The following mechanistic insights from individual studies may offer potential explanations for such bidirectional effects, should this hypothesis hold true. The core pathophysiology of sepsis-induced immune dysregulation lies in the sequential failure of innate and adaptive immunity (Hotchkiss et al., 2013). During the early stage of the disease, an over-activated innate immune response triggers a “cytokine storm,” which is the primary driver of systemic inflammatory response syndrome (SIRS). Studies have confirmed that AS can directly block the recognition signals of pathogen-associated molecular patterns (PAMPs) by specifically downregulating the transcription and expression levels of pattern recognition receptors on macrophages. This subsequently inhibits the aberrant activation of the downstream NF-κB signaling pathway, reduces IκBα phosphorylation and p65 nuclear translocation, and, ultimately, significantly diminishes the release of key pro-inflammatory cytokines such as TNF-α, IL-6, and IL-1β, thereby curbing the amplification of the inflammatory cascade (Li et al., 2008; Li et al., 2010).

As the disease progresses to the immunosuppressive phase, macrophage functional exhaustion, impaired antigen presentation capacity, and lymphocyte apoptosis lead to compromised pathogen clearance, representing the core reason for secondary infections in the late stages of sepsis (Hotchkiss et al., 2013). At this stage, AS reverses the state of immune paralysis through a dual mechanism proposed in separate reports: on the one hand, it directly binds to the vitamin D receptor (VDR) within macrophages, relieving VDR-mediated transcriptional repression of the key autophagy gene ATG16L1, thereby initiating the autophagy pathway to enhance intracellular pathogen clearance (Shang et al., 2020). On the other hand, by modulating the phosphorylation cascade of the CaMKII–CaMKKβ–AMPK–ULK1 signaling axis, it restores macrophage energy metabolism and cytokine secretion function (Liu et al., 2020). Furthermore, preliminary evidence from a study by Bang et al. suggests that AS can directly enhance the phagocytic and bactericidal/fungicidal efficiency of macrophages against intracellular bacteria and fungi by activating the G protein-coupled receptor 37 (GPR37) receptor-mediated phosphorylation of the ERK signaling pathway, creating a synergistic effect of “immunomodulation-pathogen clearance” (Bang et al., 2021). This precise targeted intervention in both the hyperinflammatory and immunosuppressive phases constitutes the core mechanism by which AS corrects sepsis-induced immune dysregulation and re-establishes systemic immune homeostasis.

#### 5.2.2 Organ protection and functional improvement

Multiple organ dysfunction syndrome (MODS) is the leading cause of mortality in sepsis (Singer et al., 2016). Our meta-analysis indicates AS’s beneficial effects on lung and liver injury markers. The following organ-specific mechanisms, primarily derived from individual studies, provide potential explanations for these protective effects. The lungs, being the primary target organ affected in sepsis, suffer from barrier dysfunction and inflammatory infiltration, which form the core pathological basis of acute respiratory distress syndrome (ARDS). Research indicates that AS can activate the Nrf2/HO-1 antioxidant signaling pathway, promoting the expression of downstream antioxidant enzymes such as superoxide dismutase (SOD) and glutathione peroxidase (GSH-Px), significantly alleviating oxidative stress injury in lung tissue. Concurrently, it inhibits neutrophil trans-endothelial migration and infiltration, reducing the intensity of the inflammatory response in lung tissue (Cao et al., 2016). Additionally, a study by Zhang et al. reported that AS can initiate the PI3K/AKT/mTOR pro-survival signaling pathway, thereby inhibiting apoptosis in alveolar epithelial cells and vascular endothelial cells, maintaining the integrity of the alveolar–capillary barrier, and reducing pulmonary edema and ventilation dysfunction (Zhang et al., 2020). Recent research in a single study further revealed that AS can upregulate the expression of the deacetylase SIRT1 and, in a SIRT1-dependent manner, inhibit the assembly and activation of the NLRP3 inflammasome, reducing caspase-1 cleavage and the maturation and release of IL-1β and IL-18, thereby mitigating pyroptosis in lung tissue and providing a novel mechanistic explanation for its lung-protective effects (Liu et al., 2023).

Regarding liver protection, a study by Yang et al. proposed that the imbalance in immune cell polarization is a key mechanism in sepsis-induced liver injury. AS downregulates the transcription level of the long non-coding RNA MALAT1, relieving its inhibitory sequestration of polypyrimidine tract binding protein 1 (PTBP1), which subsequently modulates the IFIH1-mediated innate immune signaling pathway. This promotes the Kupffer cells toward the M2 type, increases the secretion of anti-inflammatory cytokines, and suppresses hepatic inflammatory infiltration and hepatocyte damage (Yang et al., 2024). Single-cell transcriptomic studies from a specific report by Chen et al. further confirmed that AS can systematically remodel the splenic immune microenvironment in sepsis: while regulating macrophage phenotypic switching, it also restores neutrophil chemotactic function and antioxidant stress capacity, modulates the activation status of T lymphocytes (T cells) and B lymphocytes (B cells), and regulates natural killer (NK) cell cytotoxicity, thereby providing support for multi-organ protection at the systemic immune level (Chen et al., 2023).

Renal tubular epithelial cell injury and dysfunctional autophagy are central pathological links for sepsis-associated acute kidney injury (SA-AKI) (Liu et al., 2024). Research by Chen et al. indicated that AS enhances lactate clearance by promoting the expression of the lactate transporter (MCT1) in renal tubular epithelial cells. Simultaneously, it activates the AMPK signaling pathway and inhibits mTOR activity, thereby enhancing autophagic flux, accelerating the clearance of damaged organelles and toxic metabolites, maintaining the structural and functional integrity of renal tubular epithelial cells, and ultimately improving renal function indicators (Chen et al., 2025).

#### 5.2.3 Regulation of cell death and metabolism

Aberrant activation of programmed cell death and cellular metabolic disturbances are critical pathological links in sepsis-induced tissue damage and immune deficiency (van der Poll et al., 2017). Our meta-analysis supports the antiapoptotic effect of AS, as evidenced by reduced TUNEL-positive cells and an increased p-mTOR/mTOR ratio across tissues. Mechanistic insights from individual preclinical studies focused on immune and parenchymal cells offer more specific clues to how this effect is mediated through cell death and metabolic pathways. AS provides dual protection for both immune cells and parenchymal cells by precisely intervening in cell death and metabolic pathways. At the immune cell level, T-cell exhaustion and apoptosis in the late stages of sepsis are major causes of the loss of adaptive immune function. A study by Yuan et al. demonstrated that AS can downregulate the expression of inhibitory receptors (PD-1, CTLA-4) on T-cells, thus blocking the excessive inhibition mediated by immune checkpoint pathways. Concurrently, it activates the mitogen-activated protein kinase MAPK/ERK signaling pathway, reduces the activation of molecules associated with the mitochondrial apoptotic pathway, inhibits T-cell apoptosis, and maintains the body’s specific immune response capability against pathogens (Yuan et al., 2023). The results of this meta-analysis further confirm that AS universally increases the p-mTOR/mTOR ratio across multiple tissues and significantly reduces the number of TUNEL-positive cells, highlighting its consistent antiapoptotic effect across different tissues. In terms of cellular metabolism and autophagy regulation, AS exhibits distinct cell-type specificity: in immune cells, AS enhances autophagic activity to promote intracellular pathogen clearance and restore immune function (Liu et al., 2020; Shang et al., 2020), whereas in parenchymal cells, it modulates autophagy pathways to maintain intracellular homeostasis and mitigate oxidative stress and inflammatory damage (Chen et al., 2025).

#### 5.2.4 Antibacterial and detoxification effects

Beyond modulating the host immune response, preclinical evidence also suggests that AS interrupts the pathological progression of sepsis at its source through direct antibacterial and endotoxin detoxification mechanisms. Infections with drug-resistant bacteria pose a significant challenge in sepsis treatment. AS can bind with high affinity to penicillin-binding protein 2a (PBP2a) in methicillin-resistant *Staphylococcus aureus* (MRSA), inhibit its catalytic activity, and downregulate the transcription and expression of the resistance gene mecA, thereby reversing bacterial resistance to β-lactam antibiotics. This creates a synergistic “herbal medicine–antibiotic” antibacterial effect, significantly improving the efficiency of infection control (Jiang et al., 2011).

Endotoxin, a component of the Gram-negative bacterial cell wall, is the initiating factor triggering the septic inflammatory cascade (Hotchkiss and Karl, 2003). Experimental evidence indicates that AS can upregulate the expression of scavenger receptors on macrophages, enhancing the recognition, internalization, and degradation of circulating LPS, thereby reducing the peripheral blood LPS load. Simultaneously, Li et al. proposed a mechanism that it can directly inhibit the binding of LPS to the TLR4/MD-2 complex, blocking the activation of the downstream myeloid differentiation primary response 88 (MyD88)-dependent signaling pathway, thus curbing the massive release of pro-inflammatory cytokines at the source and alleviating the systemic inflammatory response (Li et al., 2014).

#### 5.2.5 Comparative advantages over other natural compounds

In comparison with other natural compounds investigated for sepsis treatment, AS demonstrates several distinctive advantages that may render it a more promising candidate. It should be noted that the comparisons are based on indirect mechanism-based analyses and findings from separate preclinical studies of each compound, as direct comparative preclinical studies evaluating these natural compounds in sepsis models are currently lacking. First, whereas the activities of resveratrol (Zhou et al., 2019) and curcumin (Zhang et al., 2025) are largely confined to anti-inflammatory and antioxidant effects, AS exhibits unique immunomodulatory properties that vary with the disease phase. It effectively suppresses the early cytokine storm while reversing immunosuppression in later stages, thereby providing a more comprehensive intervention for the biphasic immune dysregulation characteristic of sepsis. Second, unlike ulinastatin—primarily a protease inhibitor with anti-inflammatory and antiapoptotic functions—AS acts through broader mechanisms (Zhang et al., 2025). These include direct antibacterial effects, clearance of endotoxin, and precise modulation of autophagy and multiple programmed cell-death pathways. Third, AS has an established clinical safety profile owing to its extensive use in antimalarial therapy (Ampadu et al., 2018), which may facilitate its repurposing for sepsis clinical trials—an advantage that resveratrol and curcumin have yet to attain in this setting. Therefore, the multimodal and phase-aware therapeutic profile of AS not only incorporates key benefits associated with other natural compounds but also surpasses them, offering a more holistic and adaptive interventional strategy for the complex and evolving pathophysiology of sepsis. Given the current reliance on indirect comparisons, future head-to-head preclinical studies are required to directly validate the relative therapeutic efficacy and mechanistic superiority of AS against other candidate compounds in sepsis models.

### 5.3 Strengths and limitations

This study is the first systematic review to evaluate the efficacy of AS in septic animal models, providing a solid evidence base for clinical trial design and novel therapeutic development. It strictly adhered to PRISMA guidelines, used the SYRCLE tool and GRADE approach to assess methodological quality and evidence certainty respectively, registered the protocol in PROSPERO, and conducted publication bias and sensitivity analyses for key outcomes, enhancing the robustness of findings.

The limitations of this study should be considered from the following aspects. First, the reporting of critical methodological elements in the included studies, such as randomization, allocation concealment, and blinding, was generally incomplete, which may lead to an overestimation of the true treatment effect and necessitates cautious interpretation of the pooled effect estimates. The overall certainty of evidence, as reflected in the low–moderate GRADE ratings, directly stems from these methodological shortcomings. Consequently, the internal validity and, thus, the translational validity of this evidence base are constrained. Although the pooled results are promising, they should be interpreted as preliminary and hypothesis-generating, underscoring the need for future preclinical studies adhering to higher methodological standards to strengthen the evidence for potential clinical translation.

Second, substantial statistical heterogeneity (I^2^ > 50%) was observed for several critical outcomes, including bacterial load, lung injury scores, and cytokine levels. Although random-effects models and sensitivity analyses were utilized, heterogeneity constrained interpretation, and subgroup analyses were not feasible due to the limited number of studies (n < 3) per comparison. This heterogeneity predominantly stems from variations in animal species, sepsis modeling methods, and AS intervention protocols. The presence of such marked heterogeneity inherently reduces the certainty with which a single, precise treatment effect can be defined. Consequently, the point estimates for these outcomes, while suggesting a directional benefit of AS, should be interpreted with caution. They are best viewed as indicative of a potential treatment signal within a range of plausible effects rather than as precise measures of efficacy. This limitation underscores that our findings primarily highlight the need for and inform the design of more standardized future investigations. Furthermore, the considerable heterogeneity in AS dosing, route, and timing of administration across studies (Table 1) limits the comparability of the findings and complicates the direct translation of a specific therapeutic protocol. The lack of standardized protocols in preclinical studies introduces significant methodological variability, which limits the clinical relevance and translational interpretation of pooled findings. To bridge this translational gap, future research should prioritize standardized models that enhance clinical applicability. For instance, using intraperitoneal administration—the most common route in existing studies—and initiating treatment within a narrow, clinically feasible window (e.g., 1 h–4 h post-sepsis induction) would better simulate emergency intervention scenarios. Adopting such standardized, clinically aligned dosing regimens is essential to more definitively evaluate the therapeutic potential of AS and to inform the design of subsequent clinical trials.

Third, the exclusion of gray literature and reliance on published studies may have introduced publication bias. Although funnel plot symmetry and Egger’s test did not indicate significant bias, the relatively small number of studies limits the statistical power of these assessments, and thus, publication bias cannot be entirely excluded. This limitation is compounded by the substantial heterogeneity in experimental designs (e.g., dose and timing) across studies, which may itself be a source of selective reporting and could influence the magnitude and direction of the pooled effect estimates. To mitigate this issue in future research and improve the reliability of evidence synthesis, preclinical studies should aim for standardized, dose-ranging experimental designs that are more directly comparable and less susceptible to such biases. Fourth, when studies reported multiple doses or time-points, we selected the most effective dataset for synthesis. Although this strategy was intended to capture the maximal therapeutic potential of AS and facilitate a clearer synthesis, we acknowledge that it may introduce a selection bias and lead to an overestimation of the overall treatment effect. This constitutes an inherent limitation of our analysis. Additionally, although data extraction from figures was performed meticulously using standardized software, potential measurement errors cannot be entirely ruled out. Finally, as the conclusions are derived from animal studies, clinical translatability requires validation through further preclinical and clinical investigations. Nonetheless, this meta-analysis offers a systematic summary of current research in the field and provides a valuable reference to support the further development of AS for the treatment of sepsis.

## 6 Conclusion

In this study, we provide the first systematic synthesis, suggesting that AS is associated with improved survival and attenuated organ injury in septic animal models. The potential therapeutic efficacy of AS may be underpinned by a multimodal mechanism, which includes potential phase-dependent immunomodulation that counteracts both hyperinflammation and immunosuppression, coupled with antiapoptotic effects and enhanced bacterial clearance. Despite promising results, the limited number of studies and heterogeneity among studies highlight the need for more standardized preclinical research. AS is a candidate that should be further investigated for sepsis treatment.

## Data Availability

The datasets presented in this study can be found in online repositories. The names of the repository/repositories and accession number(s) can be found in the article/Supplementary Material.
