# Supplementary material for: The efficacy of artesunate in animal models of sepsis: a systematic review and meta-analysis
Source: Front Pharmacol. 2026 Jan 28;17:1748083. doi: 10.3389/fphar.2026.1748083 (PMC12891116; doi:10.3389/fphar.2026.1748083)
Supplement: Supplementary file 1 [file Supplementaryfile1.docx]

Supplementary Material

# Supplementary Tables

## Supplementary Table S1 The detailed search strategy.

| **Electronic databases** | **Search** | **Search strategy** | **Results** |
| --- | --- | --- | --- |
| **PubMed** | #1 | ((("Sepsis"[Mesh]) OR ("Systemic Inflammatory Response Syndrome"[Mesh])) OR ("Bacteremia"[Mesh])) OR( ((((((((((((((((((((((((((((((((((((Bloodstream Infection[Title/Abstract])) OR (Bloodstream Infections[Title/Abstract])) OR (Infection, Bloodstream[Title/Abstract])) OR (Septicemia[Title/Abstract])) OR (Septicemias[Title/Abstract])) OR (Blood Poisoning[Title/Abstract])) OR (Blood Poisonings[Title/Abstract])) OR (Poisonings, Blood[Title/Abstract])) OR (Poisoning, Blood[Title/Abstract])) OR (Severe Sepsis[Title/Abstract])) OR (Sepsis, Severe[Title/Abstract])) OR (Pyemia[Title/Abstract])) OR (Pyemias[Title/Abstract])) OR (Pyaemia[Title/Abstract])) OR (Pyohemia[Title/Abstract]) OR (Pyohemias[Title/Abstract])) OR (abdominal sepsis[Title/Abstract])) OR(focal sepsis[Title/Abstract])) OR(intraabdominal sepsis[Title/Abstract])) OR(sepsis syndrome[Title/Abstract])) OR(septic disease[Title/Abstract])) OR (Inflammatory Response Syndrome, Systemic[Title/Abstract])) OR (Sepsis Syndrome[Title/Abstract])) OR (Sepsis Syndromes[Title/Abstract])) OR (Syndrome, Sepsis[Title/Abstract])) OR (Syndromes, Sepsis[Title/Abstract])) OR (multi-system inflammatory syndrome[Title/Abstract])) OR (multisystem inflammatory syndrome[Title/Abstract])) OR (SIRS [Title/Abstract])) OR (systemic inflammation syndrome[Title/Abstract])) OR (systemic inflammatory syndrome[Title/Abstract])) OR (systemic inflammatory response syndrome)) OR (Bacteremias[Title/Abstract])) OR (Bacillaemia[Title/Abstract])) OR(bacillemia[Title/Abstract])) OR(bacteraemia[Title/Abstract])) OR(bacteriemia[Title/Abstract])) | 228741 |
|  | #2 | ("Artesunate"[Mesh]) OR (((((((((Sodium Artesunate[Title/Abstract]) OR (SM-804[Title/Abstract])) OR (SM 804[Title/Abstract])) OR (Malacef[Title/Abstract])) OR (Dihydroartemisinine-12-alpha-succinate[Title/Abstract])) OR (Dihydroartemisinine 12 alpha succinate[Title/Abstract])) OR (Succinyl Dihydroartemisinin[Title/Abstract])) OR (Dihydroartemisinin, Succinyl[Title/Abstract])) OR (Malartin[Title/Abstract])) OR (artesunate[Title/Abstract]) | 4182 |
|  | #3 | #1 AND #2 | **292** |
| **Embase** | #1 | 'sepsis'/exp OR 'sepsis' | 451702 |
|  | #2 | 'systemic inflammatory response syndrome'/exp | 416446 |
|  | #3 | 'bacteremia'/exp | 72389 |
|  | #4 | 'bloodstream infection':ab,ti OR 'bloodstream infections':ab,ti OR 'infection, bloodstream':ab,ti OR 'septicemia':ab,ti OR 'septicemias':ab,ti OR 'blood poisoning':ab,ti OR 'blood poisonings':ab,ti OR 'poisonings, blood':ab,ti OR 'poisoning, blood':ab,ti OR 'severe sepsis':ab,ti OR 'sepsis, severe':ab,ti OR 'pyemia':ab,ti OR 'pyemias':ab,ti OR 'pyaemia':ab,ti OR 'pyohemia':ab,ti OR 'pyohemias':ab,ti OR 'abdominal sepsis':ab,ti OR 'focal sepsis':ab,ti OR 'intraabdominal sepsis':ab,ti OR 'septic disease':ab,ti OR 'inflammat':ab,ti OR 'y response syndrome, systemic':ab,ti OR 'sepsis syndrome':ab,ti OR 'sepsis syndromes':ab,ti OR 'syndrome, sepsis':ab,ti OR 'syndromes, sepsis':ab,ti OR 'multi-system inflammat':ab,ti OR 'multisystem inflammat':ab,ti OR 'sirs':ab,ti OR 'systemic inflammation syndrome':ab,ti OR 'y syndrome':ab,ti OR 'systemic inflammat':ab,ti OR 'y response syndrome':ab,ti OR 'bacteremias':ab,ti OR 'bacillaemia':ab,ti OR 'bacillemia':ab,ti OR 'bacteraemia':ab,ti OR 'bacteriemia':ab,ti | 88665 |
|  | #5 | #1 OR #2 OR #3 OR #4 | 493777 |
|  | #6 | 'artesunate'/exp | 8441 |
|  | #7 | 'sodium artesunate':ab,ti OR 'sm-804':ab,ti OR 'sm 804':ab,ti OR 'malacef':ab,ti OR 'dihydroartemisinine-12-alpha-succinate':ab,ti OR 'dihydroartemisinine 12 alpha succinate':ab,ti OR 'succinyl dihydroartemisinin':ab,ti OR 'dihydroartemisinin':ab,ti OR 'succinyl':ab,ti OR 'malartin':ab,ti OR 'artesunate':ab,ti | 13085 |
|  | #8 | #6 OR #7 | 16804 |
|  | #9 | #5 AND #8 | **281** |
| **Cochrane Library** | #1 | MeSH descriptor: [Sepsis] explode all trees | 6604 |
|  | #2 | MeSH descriptor: [Systemic Inflammatory Response Syndrome] explode all trees | 7139 |
|  | #3 | MeSH descriptor: [Bacteremia] explode all trees | 1307 |
|  | #4 | (Bloodstream Infection or Bloodstream Infections or Infection, Bloodstream or Septicemia or Septicemias or Blood Poisoning or Blood Poisonings or Poisonings, Blood or Poisoning, Blood or Severe Sepsis or Sepsis, Severe or Pyemia or Pyemias or Pyaemia or PyohemiaorPyohemias or abdominal sepsis or focal sepsis or intraabdominal sepsis or sepsis syndrome or septic disease or Inflammatory Response Syndrome, Systemic or Sepsis Syndrome or Sepsis Syndromes or Syndrome, Sepsis or Syndromes, Sepsis or multi-system inflammatory syndrome or multisystem inflammatory syndrome or SIRS or systemic inflammation syndrome or systemic inflammatory syndrome or systemic inflammatory response syndrome or Bacteremias or Bacillaemia or bacillemia or bacteraemia or bacteriemia):ti,ab,kw | 15356 |
|  | #5 | #1 or #2 #3 or #4 | 18899 |
|  | #6 | MeSH descriptor: [Artesunate] explode all trees | 614 |
|  | #7 | (Sodium Artesunate or SM-804 or SM 804 or Malacef or Dihydroartemisinin-12α-succinate or Dihydroartemisinine 12 alpha succinate or Succinyl Dihydroartemisinin or Dihydroartemisinin, Succinyl or Malartin or artesunate):ti,ab,kw | 1256 |
|  | #8 | #6 or #7 | 1256 |
|  | #9 | #5 and #8 | **104** |
| **Web of Science** | #1 | TS=('Sepsis'OR ‘Systemic Inflammatory Response Syndrome’ OR ‘Bacteremia’ ) OR AB=(Bloodstream Infection’ OR ‘Bloodstream Infections’ OR ‘Infection, Bloodstream’ OR ‘Septicemia’ OR ‘Septicemias’ OR ‘Blood Poisoning’ OR ‘Blood Poisonings’ OR ‘Poisonings, Blood’ OR ‘Poisoning, Blood’ OR ‘Severe Sepsis’ OR ‘Sepsis, Severe’ OR ‘Pyemia’ OR ‘Pyemias’ OR ‘Pyaemia’ OR ‘Pyohemia’ OR ‘Pyohemias’ OR ‘abdominal sepsis’ OR ‘focal sepsis’ OR ‘intraabdominal sepsis’ OR ‘sepsis syndrome’ OR ‘septic disease’ OR ‘Inflammatory Response Syndrome, Systemic’ OR ‘Sepsis Syndrome’ OR ‘Sepsis Syndromes’ OR ‘Syndrome, Sepsis’ OR ‘Syndromes, Sepsis’ OR ‘multi-system inflammatory syndrome’ OR ‘multisystem inflammatory syndrome’ OR ‘SIRS’ OR ‘systemic inflammation syndrome’ OR ‘systemic inflammatory syndrome’ OR ‘systemic inflammatory response syndrome’ OR ‘Bacteremias’ OR ‘Bacillaemia’ OR ‘bacillemia’ OR ‘bacteraemia’ OR ‘bacteriemia’） | 297487 |
|  | #2 | TS=('Artesunate') OR AB=(‘Sodium Artesunate’ OR ‘SM-804’ OR ‘SM 804’ OR ‘Malacef’ OR ‘Dihydroartemisinine-12-alpha-succinate’ OR ‘Dihydroartemisinine 12 alpha succinate’ OR ‘Succinyl Dihydroartemisinin’ OR ‘Dihydroartemisinin, Succinyl’ OR ‘Malartin’ OR ‘artesunate’ ) | 7917 |
|  | #3 | (#1) AND (#2) | **79** |
| **Scopus** | #1 | TITLE-ABS-KEY ( "Sepsis" OR "Systemic Inflammatory Response Syndrome" OR "Bacteremia" OR "Bloodstream Infection" OR "Bloodstream Infections" OR "Infection, Bloodstream" OR "Septicemia" OR "Septicemias" OR "Blood Poisoning" OR "Blood Poisonings" OR "Poisonings, Blood" OR "Poisoning, Blood" OR "Severe Sepsis" OR "Sepsis, Severe" OR "Pyemia" OR "Pyemias" OR "Pyaemia" OR "Pyohemia" OR "Pyohemias" OR "abdominal sepsis" OR "focal sepsis" OR "intraabdominal sepsis" OR "sepsis syndrome" OR "septic disease" OR "Inflammatory Response Syndrome, Systemic" OR "Sepsis Syndrome" OR "Sepsis Syndromes" OR "Syndrome, Sepsis" OR "Syndromes, Sepsis" OR "multi-system inflammatory syndrome" OR "multisystem inflammatory syndrome" OR "SIRS" OR "systemic inflammation syndrome" OR "systemic inflammatory syndrome" OR "systemic inflammatory response syndrome" OR "Bacteremias" OR "Bacillaemia" OR "bacillemia" OR "bacteraemia" OR "bacteriemia" ) | 436207 |
|  | #2 | TITLE-ABS-KEY ( "Artesunate" OR "Sodium Artesunate" OR "SM-804" OR "SM 804" OR "Malacef" OR "Dihydroartemisinine-12-alpha-succinate" OR "Dihydroartemisinine 12 alpha succinate" OR "Succinyl Dihydroartemisinin" OR "Dihydroartemisinin, Succinyl" OR "Malartin" OR "artesunate" ) | 8577 |
|  | #3 | (#1) AND (#2) | **213** |

## Supplementary Table S2 Risk of Bias Summary.

| Author(year) | A | B | C | D | E | F | G | H | I | J | Total |
| --- | --- | --- | --- | --- | --- | --- | --- | --- | --- | --- | --- |
| Chen et al.(2025) | ? | ? | ? | + | ? | ? | ? | + | + | ? | 3 |
| Yang et al. (2024) | ? | ? | ? | ? | ? | ? | ? | ? | + | ? | 1 |
| Yuan et al. (2023) | + | ? | ? | + | ? | ? | ? | + | + | + | 5 |
| Liu et al. (2023) | ? | ? | ? | + | ? | ? | ? | + | + | ? | 3 |
| He et al. (2023) | ? | ? | ? | + | ? | ? | ? | + | + | + | 4 |
| Chen et al. (2023) | ? | ? | ? | ? | ? | ? | ? | + | + | + | 3 |
| Bang, S et al.（2021） | ? | ? | ? | + | + | ? | + | + | + | + | 6 |
| Zhang et al. (2020) | ? | ? | ? | + | ? | ? | ? | + | + | ? | 3 |
| Liu et al. (2020) | ? | ? | ? | + | ? | ? | ? | + | + | + | 4 |
| Shang et al. (2020) | ? | ? | ? | + | ? | ? | ? | + | + | + | 4 |
| Cao et al. (2016) | ? | ? | ? | + | ? | ? | ? | + | + | + | 4 |
| Li et al. (2014) | ? | ? | ? | ? | ? | ? | ? | + | + | + | 3 |
| Jiang et al. (2011) | ? | ? | ? | ? | ? | ? | ? | + | + | + | 3 |
| Li et al. (2010) | ? | ? | ? | ? | ? | ? | ? | + | + | + | 3 |
| Li et al. (2008) | ? | ? | ? | ? | ? | ? | ? | + | + | + | 3 |

(A) Sequence generation. (B) Baseline characteristics. (C) Allocation concealment. (D) Random housing. (E) Blinding of experimentalists. (F) Random outcome assessment. (G) Blinding of outcome assessors. (H) Incomplete outcome data. (I) Selective outcome reporting. (J) Other sources of bias. +: indicates low risk; -: indicates high risk; ?: indicates unclear risk.

## Supplementary Table S3 Quality of evidence based on GRADE’s tool.

| Outcomes | **Anticipated absolute effects^*^** (95% CI) | | Relative effect (95% CI) | № of participants (studies) | Certainty of the evidence (GRADE) |
| --- | --- | --- | --- | --- | --- |
|  | **Risk with NS** | **Risk with AS** |  |  |  |
| survival rate | 109 per 1,000 | **457 per 1,000** (318 to 603) | **OR 6.87** (3.81 to 12.41) | 348 (10 RCTs) | ⨁⨁⨁◯ Moderate^a^ |
| body weight | The mean body weight was **0** | MD **1.75 higher** (0.05 higher to 3.46 higher) | - | 22 (2 RCTs) | ⨁⨁◯◯ Low^a,b^ |
| CFU | - | SMD **1.68 lower** (3.1 lower to 0.27 lower) | - | 74 (4 RCTs) | ⨁⨁◯◯ Low^a,c^ |
| Lung injury scores | The mean lung injury scores was **0** | MD **6.97 lower** (8.98 lower to 4.97 lower) | - | 46 (3 RCTs) | ⨁⨁◯◯ Low^a,c^ |
| MPO activity | The mean MPO activity was **0** | MD **0.16 lower** (0.23 lower to 0.1 lower) | - | 32 (2 RCTs) | ⨁⨁◯◯ Low^a,c^ |
| W/D ratio | The mean W/D ratio was **0** | MD **2.68 lower** (3.76 lower to 1.6 lower) | - | 46 (3 RCTs) | ⨁⨁⨁◯ Moderate^a^ |
| AST levels | The mean AST levels was **0** | MD **113.31 lower** (144.64 lower to 81.99 lower) | - | 42 (3 RCTs) | ⨁⨁⨁◯ Moderate^a^ |
| ALT levels | The mean ALT levels was **0** | MD **56.49 lower** (85.09 lower to 27.9 lower) | - | 42 (3 RCTs) | ⨁⨁⨁◯ Moderate^a^ |
| TNF-α (up) | The mean TNF-α (up) was **0** | MD **102.16 higher** (33.06 higher to 171.27 higher) | - | 42 (3 RCTs) | ⨁⨁◯◯ Low^a,c^ |
| TNF-α (down) | The mean TNF-α (down) was **0** | MD **70.29 lower** (101.92 lower to 38.66 lower) | - | 108 (6 RCTs) | ⨁⨁◯◯ Low^a,c^ |
| IL-6 (up) | The mean IL-6 (up) was **0** | MD **422.42 higher** (99.28 higher to 745.55 higher) | - | 36 (2 RCTs) | ⨁⨁⨁◯ Moderate^a^ |
| IL-6 (down) | The mean IL-6 (down) was **0** | MD **277.42 lower** (386.12 lower to 168.71 lower) | - | 76 (4 RCTs) | ⨁⨁◯◯ Low^a,c^ |
| IL-1β (up) | The mean IL-1β (up) was **0** | MD **58.67 higher** (34.21 higher to 83.14 higher) | - | 36 (2 RCTs) | ⨁⨁⨁◯ Moderate^a^ |
| caspase-3 expression | The mean caspase-3 expression was **0** | MD **3.17 lower** (8.74 lower to 2.39 higher) | - | 36 (2 RCTs) | ⨁◯◯◯ Very low^a,c,d^ |
| TUNEL positive cells | The mean TUNEL positive cells was **0** | MD **43.8 lower** (50.17 lower to 37.43 lower) | - | 32 (2 RCTs) | ⨁⨁⨁◯ Moderate^a^ |
| p-mTOR/mTOR ratio | The mean p-mTOR/mTOR ratio was **0** | MD **0.61 higher** (0.46 higher to 0.77 higher) | - | 26 (2 RCTs) | ⨁⨁◯◯ Low^a,c^ |

#### Explanations

a. high risk of bias from selecting the reported results and carryover effects; b. small sample size; c. high heterogeneity; d. 95% CI fails to exclude important benefit or important harm

# Supplementary Figures


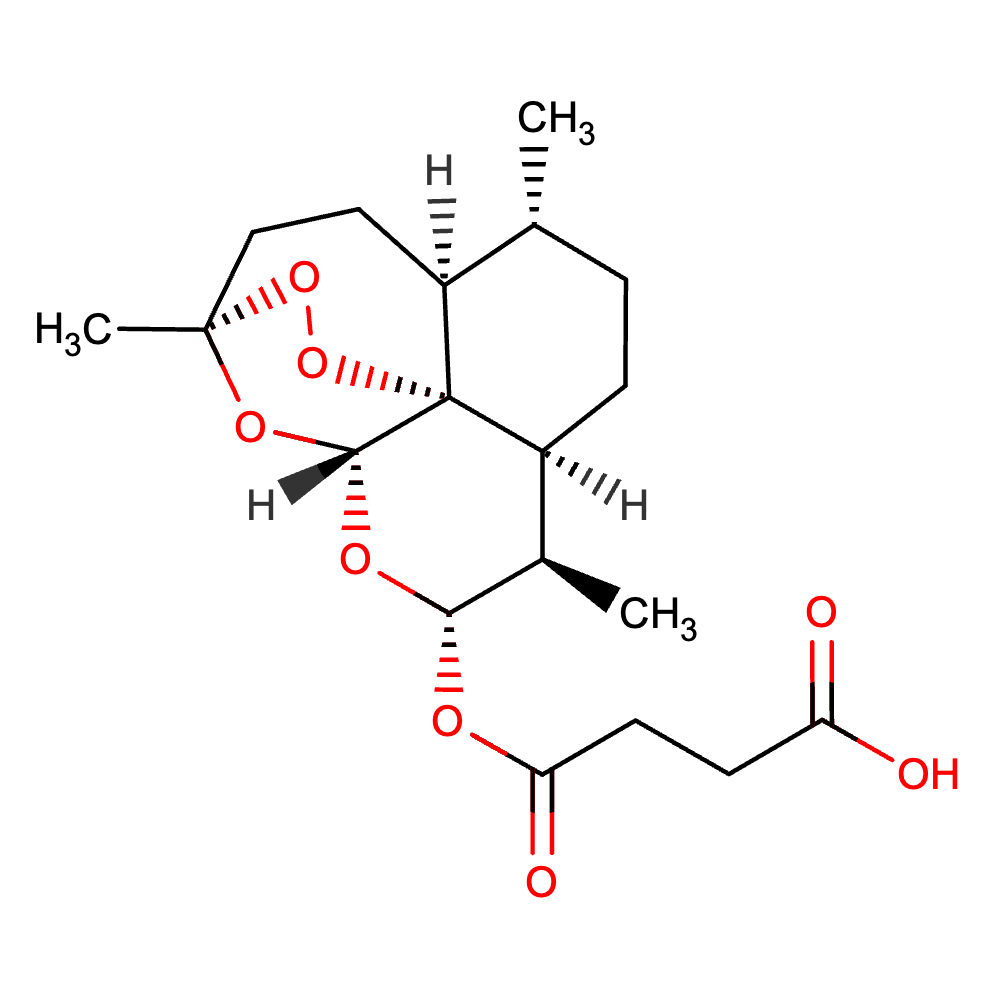


## Supplementary Figure 1. Artesunate chemical structure from DRUGBANK Online (https://go.drugbank.com/drugs/DB09274).


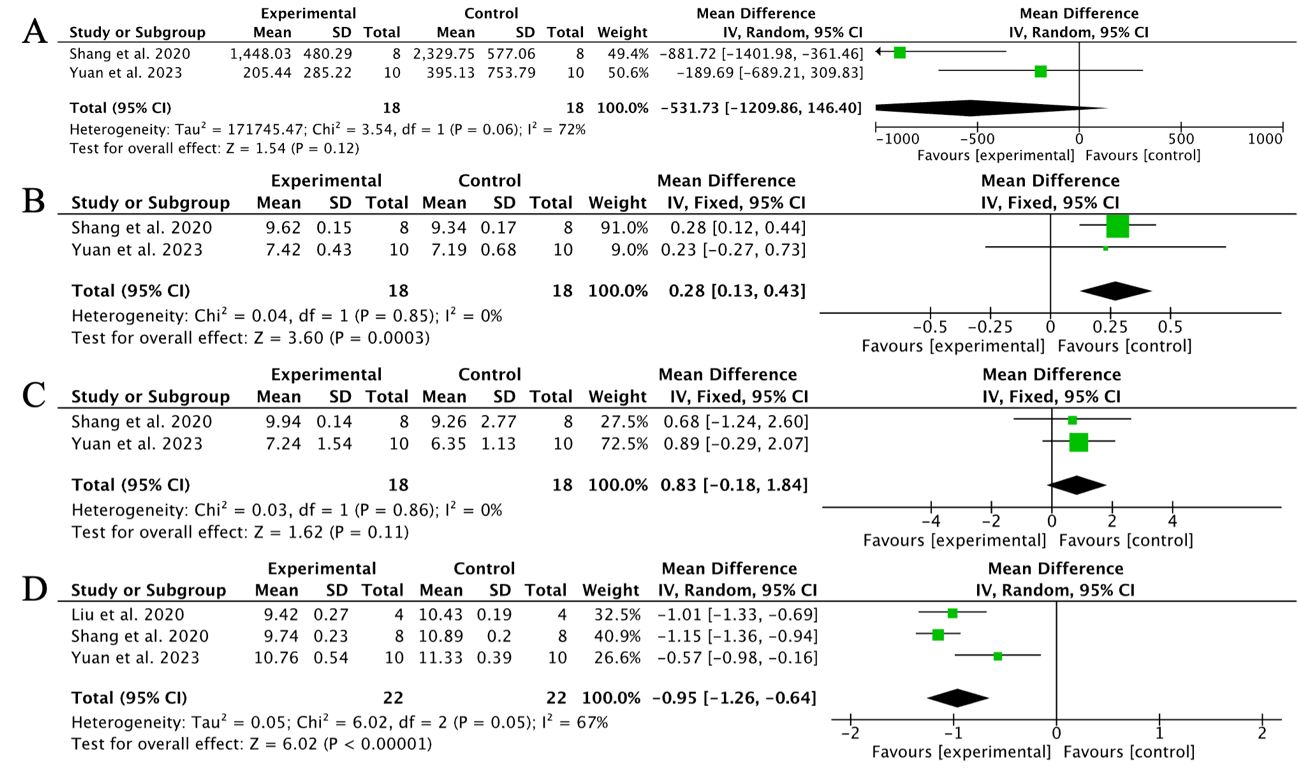


## Supplementary Figure 2. Forest plots of the effects of artesunate vs control on spleen. (A) TNF-α (down), (B) IL-6 (up), (C) IL-1β (up) and (D) CFU.


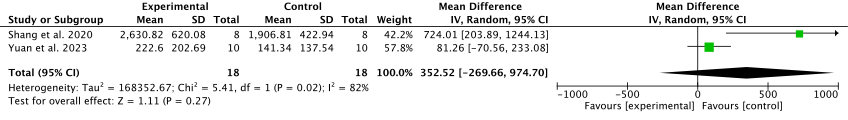


A


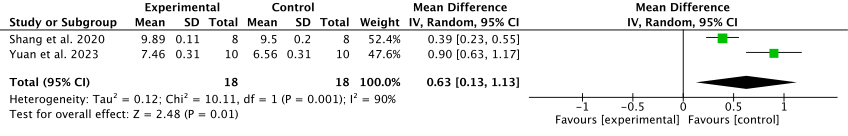


C


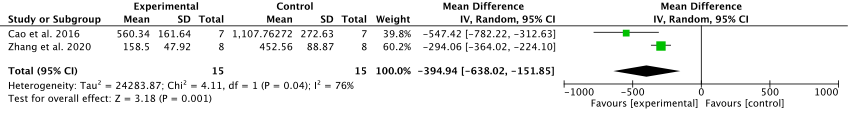


D


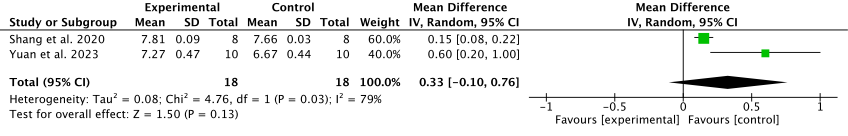


E


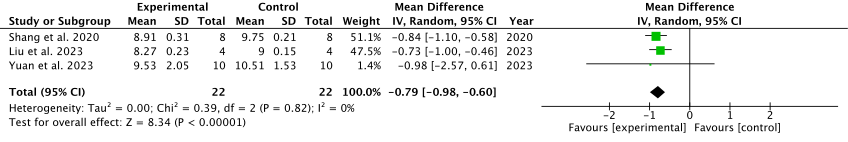


F


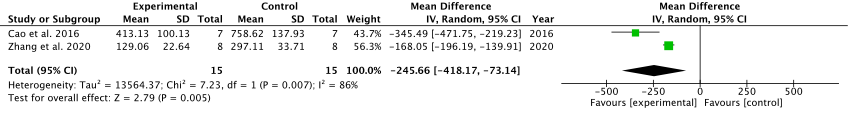


B

## Supplementary Figure 3. Forest plots of the effects of artesunate vs control on lung. (A) TNF-α (up), (B) TNF-α (down), (C) IL-6 (up), (D) IL-6 (down), (E) IL-1β (up) and (F) CFU.
